# Pesticides and pharmaceuticals data collected during two consecutive years in a Mediterranean micro-estuary

**DOI:** 10.1016/j.dib.2023.109456

**Published:** 2023-07-28

**Authors:** Tom Topaz, Julius Ben-Ari, Evgenia Kertsnus Banchik, Or Bassa, Roey Egozi, Yair Suari, Tal Sade, Hadar Zedaka, Merav Gilboa, Gitai Yahel, Benny Chefetz

**Affiliations:** a Faculty of Marine Sciences, Ruppin Academic Center, Mikhmoret, 402970, Israel; b The Interdepartmental Analytical Unit, Robert H. Smith Faculty of Agriculture, Food and Environment, The Hebrew University of Jerusalem, Rehovot, 7610001, Israel; c Dept. of Soil and Water Sciences, Robert H. Smith Faculty of Agriculture, Food and Environment, The Hebrew University of Jerusalem, P.O. Box 12, Rehovot, 7610001, Israel; d The Soil Erosion Research Station, Soil Conservation and Drainage Division, Ministry of Agriculture and Rural Development, Bet Dagan, 50250, Israel

**Keywords:** Pollutants, Baseflow, Stormwater, Environment

## Abstract

The Alexander micro-estuary, located at the eastern edge of the Mediterranean Sea, is a typical example of small water bodies that suffer from a combination of urban and agricultural pollution, and overuse of its natural water sources. It is∼6.5 km long, with maximum depth of 3 m and maximum width of 45 m. To evaluate the anthropogenic stress on the system and its ability to mitigate pollution, water samples were collected within the framework of Ruppin's Estuarine and Coastal Observatory [1]. Water samples were collected from the estuary head, which drains about 510 km^2^, and at a point 300 m upstream from the estuary mouth before water flows into the Mediterranean Sea. A total of 236 stormwater and 44 base-flow water samples between December 2016 and December 2018. Stormwater samples were collected every 0.25 – 4 h along the entire course of the flow events using an automated samplers (Sigma 900, Hach Company, Loveland CO, USA; and ISCO 3700 Full-Size Portable Sampler, Teledyne, Lincoln, NE, USA). Base-flow samples were taken once a month using a horizontal grab sampler (5 L, model 110B, OceanTest Equipment, Fort Lauderdale, FL, USA). All samples were filtered using 90mmGF/F filters (nominal pore size of 0.7 µm, MGF, Sartorius, Göttingen, Germany) and immediately frozen (−20°C) before chemical analysis. Chemical analysis was performed using liquid chromatography with high-resolution mass spectrometry (LC–HRMS) analysis using a QExactive Plus hybrid FT mass spectrometer coupled with a Dionex Ultimate 3000 RS UPLC (Thermo Fisher Scientific, Waltham, MA, USA). The targeted analysis, which included 15 fungicides, 25 herbicides, 18 Insecticides, and 19 pharmaceuticals, concluded with a total of 21,142 entries. All entries are organized in a worksheet, along with location, date, flood section duration, discharge rate, and the total water volume discharged during the relevant period. The provided data offers an opportunity to explore the sources, transport, and impact of a large mixture of organic pollutants in a confined aquatic system located in an urbanized coastal environment.

Specifications Table

**Table:** 

Subject	Environmental Chemistry, Pollution, Hydrology and Water Quality, Water Science and Technology.
Specific subject area	Occurrence and concentrations of organic pollutants in aquatic systems.
Type of data	Table
How the data were acquired	During baseflow conditions, samples were collected once a month using a horizontal water sampler (5L, Model 110B, OceanTest Equipment). During flood events, samples were collected once every 0.25 to 4 hours using an automated sampler (Sigma 900©, Hach Company, Loveland, CO) equipped with a carousel that holds 24 glass bottles (350 mL). Sampling intensity was highest during the rising limb of the hydrograph and at peak discharge, decreasing along the lowering limb and flood's tail. Water samples were analyzed using liquid chromatography high-resolution mass spectrometry. 200 mL aliquots were spiked with 10 µL of a mixture of isotopically labeled internal standards (see the detailed information in the Supporting Information, SI) and concentrated using SPE cartridges (Strata-X, 200 mg, Phenomenex, Torrance, CA). Quantification was done using liquid chromatography with high-resolution mass spectrometry (LC−HRMS) analysis using a QExactive Plus hybrid FT mass spectrometer coupled with a Dionex Ultimate 3000 RS UPLC (Thermo Fisher Scientific, Waltham, MA).
Data format	Raw
Description of data collection	Water samples were collected during baseflow and flood events along the hydrological years of 2017 and 2018. Baseflow was composed of natural flow mixed with treated wastewater. Flood events were composed of stormwater runoff from agriculture and urban regions. Data is raw and un-normalized, presenting organic pollutants concentrations as retrieved from mass spectrometry analysis. Below is the data as arranged in the repository including the headers and two example rows:
	
	
	Columns description: **Column A**, Flow Type: water sample was taken during flood event or baseflow. **Column B**, Event tag: represents the type of flow (F flood, B Baseflow), first two digits indicates the year (16 – 2016, 17 – 2017, 18 – 2018), and last digit the chronological location of the event (1 – first flood, 2- second flood, etc.).
	**Column C**, Station: A0, Head of the estuary, A4, Mouth of the estuary. **Column D**, Sample: sample ID number. **Column E**, Date: the date the sample was taken. **Column F**, Time: the time the sample was taken. **Column G**, Section duration: the time span that the sample represents. **Column H**, Discharge: the average discharge measured for the time span that the sample represents. **Column I**, Section volume: the total water volume calculated for the time span that the sample represents. **Columns J-CH**, Pollutants: the measured concentration of pollutants in water sample in µg l^−1^.
Data source location	Institution: Faculty of Marine Sciences, Ruppin Academic Center, Michmoret, Israel City/Town/Region: Emek Hefer Country: Israel
	
Data accessibility	The dataset was uploaded to the PANGAEA repository and can be accessed by this DOI –https://doi.pangaea.de/10.1594/PANGAEA.956766
Related research article	[2] Attenuation of organic pollutants and the effects of salinity and seasonality in a Mediterranean micro-estuary, Science of The Total Environment, Volume 856, Part 1, 2023, 158919, ISSN 0048-9697, https://doi.org/10.1016/j.scitotenv.2022.158919

## 1 Value of the Data

• Complex mixtures of organic pollutants have become ubiquitous in aquatic systems, imposing a growing concern for the environment and human health.
• Micro-estuaries (estuaries with a surface area of less than one square kilometer) are very abundant, especially in the highly populated coastal areas of the world.
• The documented water body that suffers from a lack of natural water and baseflow mixed with effluents, is a common state in semi-arid areas and is expected to become more abundant due to global warming and increased water utilization in more temperate zones, such as central Europe.
• This is the first published dataset of organic pollutants occurrence and concentrations in a micro-estuary along an annual cycle.
• The data joins the growing knowledge on organic pollutants in the environment [3] and can be used to compare pollution with other systems, explore risk potential, examine temporal changes in organic mixture composition, implement toxicological and other lab results to field data, and further investigate transport, fate, and impact of organic pollutant mixtures in aquatic habitats.
• The data can be used for additional multi-disciplinal science, and for the implementation of regulation and policy with the aim of mitigating environmental pollution.

## 2 Objective

Micro-estuaries are small water bodies, often located in highly populated coastal areas, suffering from point and non-point pollution. The occurrence and dynamics of Complex mixtures of organic pollutants, imposing a growing concern for the environment and human health, was completely missing from the literature for these ubiquitous water bodies. Therefore, the main objective of the presented work was to establish a dataset tracking the spatial and temporal occurrence of multiple organic pollutants. So far, the data has enabled to identify pollution sources, track seasonal dynamics, explore potential ecological risk, and evaluate the functioning of the micro-estuary as a biofilter [2,4,5].

## 3 Data Description

All data are located in a single worksheet. The database presents concentrations of organic pollutants (columns I-CH) measured during two consecutive years at the head and mouth of the Alexander micro-estuary, Israel. Metadata (columns A-I) includes flow type (baseflow, flood event), the event ID code (B for baseflow, F for flood, two first digits for year, and the following digit for the order of the event within that year), Station (A0 for estuary head, A4 for estuary mouth, see [1]), Sample number, Date, Hour, the measured section duration (in minutes), Average discharge for the section measured (in m^3^ sec^−1^), and the section volume (duration multiplied by discharge, in m^3^). Columns J-CH present pollutants concentrations in µg L^−1^.

## 4 Experimental Design, Materials and Methods

### 4.1 Study site

**Fig. 1:**
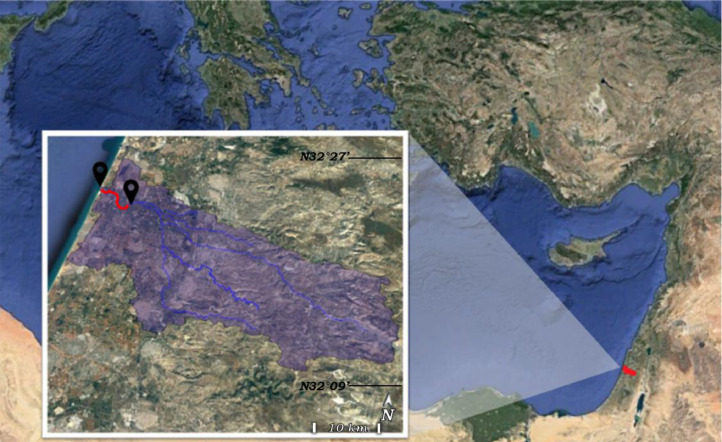
Sampling points (black locators) at the head and mouth of the Alexander micro-estuary (red line). Taken from Topaz T (2022).

The Alexander stream main channel flows a distance of 32 km and drains an area of 550 km^2^. It starts in the Samaria mountains (Palestinian Territory), crosses the Hefer valley (Israel), and ends at the Mediterranean Sea (Fig. 1). The Alexander stream is ephemeral throughout most of its length, receiving some spring water and mainly poorly treated wastewater from a treatment facility located ∼13 km upstream from the estuary head. The Alexander micro-estuary is ∼6.5 km long with a maximal depth of ∼3 m and an average cross-sectional width of 20 m. For more details about the micro-estuary, its hydrology, hydrography, and geochemistry see Suari et al. [6]. The dynamics and toxicity of pollutants in the system were described by Topaz et al. [1,2,6]. A comprehensive database that contains in situ stationary sensors data (10 min intervals) of surface and bottom temperature, salinity, oxygen, and water level measured at three stations along the estuary, monthly CTD profiles, and discrete biogeochemical samples (surface and bottom water) of multiple biogeochemical parameters at four stations along the estuary is provided by Ruppin's Estuarine and Coastal Observatory (RECO), see [1].

### 4.2 Sampling

Surface water upstream from the estuary head (N32.375 E34.912) and adjacent to the estuary mouth (N32.394 E34.869) was sampled at the depth of ∼20 cm. Each of the sampling stations was equipped with an automated water sampler (Sigma 900, Hach Company, Loveland CO; and ISCO 3700 Full-Size Portable Sampler, Teledyne, Lincoln NE) with a carousel containing 24 glass bottles of 350 mL. During flood events, samples were taken every 0.25−4 h, with a higher sampling frequency during the rising limb of the hydrograph and peak discharge and a lower sampling frequency on the falling limb of the hydrograph. During base-flow, water grab samples were collected once a month with a horizontal water sampler (5 L, model 110B, OceanTest Equipment, Fort Lauderdale FL). All samples were filtered using 90 mm GF/F filters (nominal pore size of 0.7 µm, MGF, Sartorius, Göttingen, Germany) and immediately frozen (−20°C). A total of 236 flood samples and 44 base-flow samples were analyzed over 2 hydrological years (2016−2018).

### 4.3 Analysis of organic pollutants

Water samples were defrosted overnight and 200 mL aliquots were spiked with 10 µL of a mixture of isotopically labeled internal standards and concentrated using SPE cartridges (Strata-X, 200 mg, Phenomenex, Torrance, CA). pollutants were quantified by liquid chromatography with high-resolution mass spectrometry (LC−HRMS) analysis using a QExactive Plus hybrid FT mass spectrometer coupled with a Dionex Ultimate 3000 RS UPLC (Thermo Fisher Scientific, Waltham, MA). Additional details of instrumental parameters, limit of quantification, and recoveries can be found elsewhere [4,5]. All identified and quantified pollutants were arranged in an excel sheet along with metadata and additional information.

## Ethics Statements

No ethical statements are required for the data collected.

## CRediT authorship contribution statement

**Tom Topaz:** Conceptualization, Methodology, Data curation, Project administration, Writing – original draft. **Julius Ben-Ari:** Formal analysis, Writing – review & editing. **Evgenia Kertsnus Banchik:** Formal analysis. **Or Bassa:** Data curation. **Roey Egozi:** Supervision, Conceptualization, Methodology, Writing – review & editing. **Yair Suari:** Data curation, Writing – review & editing. **Tal Sade:** Data curation. **Hadar Zedaka:** Data curation. **Merav Gilboa:** Data curation. **Gitai Yahel:** Supervision, Conceptualization, Methodology, Writing – review & editing. **Benny Chefetz:** Supervision, Conceptualization, Methodology, Writing – review & editing.

## Data Availability

Pesticides and pharmaceuticals data collected during two consecutive years in a Mediterranean micro-estuary (Original data) (PANGAEA).
